# Coexistence of methanogenesis and sulfate reduction in a sulfate-adapted enrichment culture from an oil reservoir

**DOI:** 10.1128/aem.00141-25

**Published:** 2025-11-13

**Authors:** Sebastian Beilig, Lisa Voskuhl, Itır Geydirici, Lucie K. Tintrop, Torsten C. Schmidt, Rainer U. Meckenstock

**Affiliations:** 1 Environmental Microbiology and Biotechnology, Aquatic Microbiology, University of Duisburg-Essen 39081, Essen, Germany; 2 Environmental Microbiology and Biotechnology (EMB), Microbiology of Ecotones, University of Duisburg-Essen 27170, Essen, Germany; 3 Instrumental Analytical Chemistry, University of Duisburg-Essen, Essen, Germany; 4 Centre for Water and Environmental Research, University of Duisburg-Essen, Essen, Germany; Universidad de los Andes, Bogotá, Colombia

**Keywords:** archaea, sulfate reduction, community composition, 16S rRNA gene, hydrocarbon degradation, coexistence

## Abstract

Oil reservoirs are complex ecosystems where microorganisms play a vital role in hydrocarbon degradation, mostly with methanogenesis as the terminal electron-accepting process. Especially in offshore oil reservoirs, sulfate-containing seawater is injected during the oil production to maintain the reservoir pressure, resulting in increased sulfate reduction in the reservoir. Methanogenesis and sulfate reduction are typically thought to be mutually exclusive because sulfate reducers outcompete methanogens thermodynamically for hydrogen and acetate. However, coexistence is evident in environments like landfills, estuaries, marine, and polluted river sediments. Here, we studied methanogenesis in an adapted sulfate-reducing microbial community, enriched from an oil reservoir, using incubation experiments with metabolic inhibitors to assess microbial activity and degradation potential. The observed methane production rate accounted for 156.9 µM/a with a parallel carbon dioxide production rate of 67 mM/a and a sulfate reduction rate of 20 mM/a, suggesting a coexistence of sulfate reduction and methanogenesis even in a well-mixed system. The microbial community was predominantly composed of potential sulfate-reducing bacteria including the genera *Desulfotignum*, *Desulfospira*, and *Geoalkalibacter*. In addition, fermentative bacteria such as *Mesotoga* and *Petrotoga* were abundant and the methanogenic genera *Methanolobus* as well as the order *Candidatus* Methanofastidiosales were most prevalent among the archaea. These study results suggest that even with sulfate-adapted communities and high sulfate concentrations methanogenesis can co-occur to minor extents.

**IMPORTANCE:**

This study demonstrates the coexistence of two microbial processes—methanogenesis (methane production) and sulfate reduction—in sulfate-rich environments using a microbial community derived from an oil reservoir. Conventionally, these processes were considered mutually exclusive, as sulfate-reducing microbes are thought to outcompete methanogens for shared substrates. However, this research reveals that methane production can persist alongside active sulfate reduction, challenging established paradigms of microbial competition and metabolic exclusivity.

## INTRODUCTION

Oil reservoirs are intricate ecosystems where microorganisms play a crucial role in altering and transforming hydrocarbons. In shallow reservoirs, aerobic degradation can occur, but anaerobic degradation dominates in deeper subsurface environments due to the rapid depletion of molecular oxygen (1). Since other dissolved or solid electron acceptors, such as nitrate, sulfate, or ferric iron, are depleted in oil reservoirs, fermentation and methanogenesis are considered the main processes responsible for oil degradation in most reservoirs (2, 3). The removal of n-alkanes, which are often the first compounds to be degraded in oil reservoirs, generates methane primarily through hydrogenotrophic and acetoclastic methanogenesis (4). Heavily biodegraded oil fields are often associated with methane-rich gases, originating from the methanogenic degradation of hydrocarbons (4, 5). While hydrocarbon degradation in oil reservoirs relies primarily on fermentation and methanogenesis due to the depletion of alternative electron acceptors, seawater injection during oil production introduces new oxidants such as sulfate (3). This influx can stimulate sulfate-reducing microorganisms, which compete with methanogens for common substrates, such as hydrogen and acetate. Such interactions between sulfate reducers and methanogens were studied in well-mixed anaerobic digesters, where the hindrance of methanogenesis directly impacted the energy yield and economic viability. However, such interactions remain relatively underexplored in the context of oil reservoirs (6, 7). The competition is generally thought to be dominated by sulfate reducers, since they achieve a higher thermodynamic energy yield and can deplete hydrogen and acetate to lower residual concentrations (8–13). Under standard conditions, both acetotrophic (ΔG'_o_ = −47.6 kJ/mol) and hydrogenotrophic (ΔG'_o_ = −192.0 kJ/mol) sulfate reduction are energetically more favorable than acetoclastic (ΔG'_o_ = −31.0 kJ/mol) and hydrogenotrophic (ΔG'_o_ = −175.4 kJ/mol) methanogenesis (14). In addition to thermodynamics, substrate affinity and uptake kinetics also play an important role in the competition between sulfate reducers and methanogens. Sulfate reducers have lower half-saturation constants (K_m_-values) for hydrogen (1–5 nM) and acetate (10–70 µM), while methanogens often require higher substrate concentrations >7–10 nM for hydrogen and >100 µM for acetate to maintain growth and metabolic activity (15–18). Consequently, the better substrate uptake and higher energy yield by sulfate reducers promote their growth, outcompeting methogens (19–22). This led to the common assumption that methanogenesis cannot occur in parallel with sulfate reduction.

Furthermore, direct inhibition of methanogenesis can arise from the toxicity of sulfide to various microbial groups, including methanogens and sulfate-reducing bacteria themselves (14, 23–26). The sulfide produced during sulfate reduction can hinder methanogenesis at sulfide concentrations from 2.6 to 7.3 mM, depending on the pH (27). Nevertheless, co-occurrence of methanogenesis and sulfate reduction has been observed in marine (28, 29) and estuarine (30) sediments. This co-occurrence of methanogenesis may be attributed to non-competitive substrate uptake of specific compounds, such as methanol, methanethiol, and methylamines (20, 31). Alternatively, a cooperative interaction could exist between acetogenic sulfate reducers and hydrogenotrophic methanogens (32). However, not all methanogens necessarily compete with sulfate reducers for hydrogen or acetate. Recent studies showed that the methanogenic archaeon *Candidatus* Methanoliparum is capable of complete long-chain alkane degradation coupled to methanogenesis without a syntrophic, fermenting partner (33–35).

Despite the insights into the coexistence of sulfate reduction and methanogenesis, the factors influencing this relationship remain mostly unclear, especially for oil reservoirs. Here, we aimed at understanding the competition or cooperation between sulfate reduction and methanogenesis when crude oil is used as the sole carbon and electron source. We examined a sulfate-reducing, oil-degrading microbial community enriched from an offshore oil reservoir in Trinidad and Tobago. The enrichment culture was maintained with a high sulfate concentration of 20 mM for 4 years prior to the experiment and was clearly adapted to sulfate reduction. By comparing the oil mineralization rates of specifically inhibited microbial communities, we aimed at elucidating the competition between sulfate reducers and methanogens in the context of oil degradation.

## MATERIALS AND METHODS

### Microcosm experiment setup

Microbial incubations were prepared in 120 mL serum bottles, each receiving 1 mL of autoclaved light oil (see below). A volume of 45 mL of anoxic saltwater medium (NaCl 10 g/L, MgCl_2_ 3 g/L, Na_2_SO_4_ 1.42 g/L, KCl 0.5 g/L, NH_4_Cl 0.3 g/L, KH_2_PO_4_ 0.2 g/L, CaCl_2_ 0.15 g/L) was added to each bottle (36). The serum bottles, media, and light oil were autoclaved separately and put together under sterile conditions. The inoculum had sulfate concentrations of 20 mM; no vitamins or trace metals were added to the incubations. The bottles were flushed with nitrogen/carbon dioxide (80/20 vol/vol%, Air Liquide, Düsseldorf, Germany) and sealed with butyl rubber stoppers (Ochs Laborbedarf, Bovenden, Germany).

The medium was buffered with ^13^C-labeled bicarbonate (10 atom percent ^13^C) to a final concentration of 10 mM. This was achieved using a mixture of ^13^C-labeled NaHCO_3_ (98% ^13^C-atom percent, Sigma-Aldrich, MO, USA) and regular NaHCO_3_ (1.11% ^13^C-atom percent, Carl Roth, Germany). Inert buffers like HEPES or MOPS were not used, because the reverse stable isotope labeling (RSIL) method relies on the bicarbonate–CO₂ equilibrium, enabling isotopic analysis of ¹³CO_2_/^12^CO_2_ ratios. To eliminate any remaining oxygen, 0.5 mM of Na_2_S was added, as described by Widdel and Pfennig (37), 10 mM sodium sulfate was added as an electron acceptor.

For inoculation, we used a sulfate enrichment culture (10% vol/vol) originally obtained from an offshore oil reservoir near Trinidad, Tobago, with the most abundant phyla being Desulfobacterota, Thermotogota, Bacteroidota, Bacillota (formerly Firmicutes), and Synergistota (38). This enrichment culture has previously been cultivated with 20 mM sulfate for almost 3.8 years. The experimental design comprised three sets of biological triplicates, one without inhibitors, one with 20 mM 2-bromoethanesulfonate (BES) (39, 40) to inhibit methanogenesis, and one with 10 mM sodium perchlorate (29, 41, 42) to inhibit sulfate reduction. Due to the ineffectiveness of sodium perchlorate as an inhibitor, 1 mM sodium molybdate (29, 43) was added in addition after 32 days of incubation.

The cell density at the start of the experiment was determined using a NovoCyte flow cytometer (Agilent, CA, USA, conditions explained in paragraph Monitoring of cell growth) with 11.5 × 10^5^ ± 8.4 × 10^4^ cells/mL (control), 11.7 × 10^5^ ± 1.2 × 10^5^ cells/mL (perchlorate/molybdate), and 14.1 × 10^5^ ± 8.3 × 10^4^ cells/mL (BES) obtained from the same inoculum.

### Oil source

The crude oil used as the sole carbon source in the microcosms originated from an oil field located in the central North Sea, approximately 20 km east of the Frigg field, specifically from well 25/2-18A located at 59°49'30.11'' N and 2°37'54.1'' E. The reservoir consists of Middle Jurassic sandstone from the Hugin Formation, found at a depth of 3,500 m below the seafloor, which is at 120 m water depth. The oil extracted from this field is composed of 57.5% decanes and heavier hydrocarbons, 7.2% nonanes, 11.6% octanes, and 10.2% heptanes. The remaining fraction consists of hexanes and lighter hydrocarbons. (personal information, Erling Rykkelid, AkerBP, Lysaker, Norway).

### Monitoring of cell growth

Immediately following each sampling point, cell growth was monitored using a NovoCyte flow cytometer (Agilent, CA, USA) utilizing a 488 nm FITC laser. For this, a 500 µL sample was diluted 1:2 in ultra-pure water (0.05 µS/cm, TOC < 5 ppb, Merck Millipore, Darmstadt, Germany). Samples were filtered through 0.45 µm membranes (Filtropur S, Sarstedt, Nümbrecht, Germany) in order to prevent the flow cytometer capillary from clogging by larger oil micelles. The samples were diluted in ultra-pure water to achieve <2,000 events/s during measurement. A dye mixture of SYBR Green (10,000× concentrate in DMSO, 1× end concentration) and propidium iodide (40 µM end concentration) was prepared. The dye mixture was added to 200 µL of the sample in a U-bottom 96-well plate and incubated in the dark for 13 min at room temperature. Measurement was conducted at a 14 µL/mL flow rate and a fixed sample volume of 50 µL. Cells were counted using a custom gate applied to the green fluorescence signal (488 nm), with the threshold set at 1,000. The instrument was calibrated in advance with NovoCyte QC Particles (Agilent Santa Clara, USA).

### 16S rRNA gene sequence community analysis

To extract DNA from the samples, 2 mL of each microcosm was filtered through 0.2 µm filters (Isopore 0.2 µm GTBP, Merck Millipore, Tullagreen, Ireland) under sterile conditions. Entire filters were subsequently transferred into bead-beating tubes (PowerBead Tubes, Qiagen). DNA extraction was performed using a combination of phenol-chloroform extraction and the DNeasy PowerLyzer PowerSoil kit (Qiagen), following previously established protocols (38, 44). For method validation, both positive and negative controls were treated identically to the samples. The negative control consisted of DNA-free water, while the positive control contained a commercial Mock community (ZymoBIOMICS Microbial Community Standard, Zymo Research Europe, Freiburg, Germany). The extracted DNA was purified and concentrated using magnetic beads (MagSi-NGSPREP Plus, magtivio) and adjusted to a final volume of 30 µL (Illumina Innovative Technologies Inc., 2013).

The V4 region of the bacterial and archaeal 16S rRNA genes was targeted using the primer set S-D-Arch-0519-a-S-15 (5′-CAGCCGCCGCGGTAA-3′) and S-D-Bact-0785-b-A-18 (5′-GACTACHVGGGTATCTAATCC-3′) through touchdown PCR (45, 46). The touchdown PCR protocol was carried out as previously described (47).

Amplicon PCR products were prepared for ready-to-load sequencing by the company CeGat GmbH (Tübingen, Germany) as previously described (38). Sequencing was performed by an Illumina MiSeq system V2 with 500 cycles of 250 base pair paired ends.

Raw sequencing reads were processed using the DADA2 (version 1.24.0) and phyloseq (version 1.40.0) pipelines in R (48, 49). Due to the low sequencing quality of the reverse reads and resulting trimming, there was no overlap sequence with the forward reads anymore, and only the forward reads have been employed for further analysis and were trimmed to 200 base pairs (filterAndTrim(truncLen = 200, maxN = 0, maxEE = 2, truncQ = 2)). The use of only forward reads may limit the taxonomic resolution of the data set, particularly for closely related taxa, such as certain archaeal lineages. Taxonomy was assigned using the SILVA database (version 138.1), and the sequences were clustered into amplicon sequence variants (ASVs) (48, 50, 51). Before data analysis, all taxa with reads below 10 over all samples were removed from the data set, as considered as contamination. Additionally, all reads classified as chloroplasts and mitochondria were removed from the data set.

### Microbial activity measurements via reverse stable isotope labeling method

Oil degradation rates were determined using the RSIL method. This approach uses ¹³C-labeled bicarbonate as a buffer to establish a defined δ¹³C value in the dissolved inorganic carbon pool, not as a carbon source. If the supplied substrate (oil) is biodegraded to CO₂, the δ¹³C signal becomes diluted due to the lower natural abundance of ¹³C in the oil (~1.1%). Since the ¹³C concentration in the microcosm is known and stable, the amount of CO₂ produced from oil mineralization can be quantified via isotope balance calculations based on shifts in the δ¹³C ratios as published before (38, 52, 53). For each sampling point, 3 mL microcosm liquid was sampled with a nitrogen-flushed syringe through the stopper of each microcosm under sterile conditions.

The bacterial oil mineralization to carbon dioxide was monitored by RSIL as described before (38). Samples of the aqueous phase (1 mL) were transferred into 12 mL Labco exertainer vials (Labco Limited, UK) containing 50 µL of 85% phosphoric acid. The vials had been sealed with butyl rubber septa and screw caps and purged with carbon dioxide-free synthetic air (6.0 grade; Air Liquide, Germany) prior to sampling (52–55).

The samples were subsequently analyzed with a Delta Ray CO_2_ Isotope Ratio Infrared Spectrometer (Thermo Fisher Scientific, MA, USA) equipped with a Universal Reference Interface. To ensure the highest level of precision, the carbon dioxide concentration of both the reference (Carbon Dioxide N45, Air Liquide, Düsseldorf, Germany) and sample gas entering the machine was set at 380 ppm. After a 5-min measurement for each sample, the resulting δ^13^C values, indicating the isotope ratio of ^13^C to ^12^C, were averaged and converted into isotope-amount fraction (x(^13^C) CO_2_) as reported before (38, 56). The isotope amount fraction indicates the proportion of ^13^C present in the gas phase derived from dissolved inorganic carbon in the aqueous phase, which volatilizes through the acid. The Vienna PeeDee Belemnite standard ratio needed for the calculations provides a benchmark for stable carbon isotope ratios, defined as δ^13^C = 0.0111802. To express the isotope amount fraction in atom percent, it was multiplied by 100 (atom percent = x(^13^C)CO_2_ × 100 [%]). This calculation reflects the percentage abundance of ^13^C atoms in the sample and serves as the basis for determining the amount of carbon dioxide produced (38). All calculations utilized the actual total carbon dioxide concentration of 5.9 mM present in the buffer, along with a ^13^C content of 1.075% in the substrate oil (44).

pH values were monitored throughout the entire experiment and remained stable between 7.5 and 8. The carbonate equilibrium (57) states that in this pH range, the majority of the carbon dioxide produced was fully dissolved as bicarbonate in the liquid phase, with only a negligible amount present in the gas phase, ensuring accurate measurements.

### Sulfate reduction and sulfide evolution

The concentration of the terminal electron acceptor sulfate was measured using a Dionex Aquion ion chromatography (Thermo Scientific, USA). Organic compounds were extracted from the samples through solid-phase extraction, utilizing octadecyl-modified silica gel cartridges (Chromabond 1 mL, 100 mg sorbent, Macherey-Nagel, Düren, Germany). The sorbent was conditioned with methanol, after which the samples were filtered and prepared for analysis as described in Voskuhl et al. (58).

To determine the sulfide concentration via spectrophotometric assays, zinc acetate was added to a 20 µL sample to achieve a final concentration of 47.6 mM. The samples were vortexed, and three 100 µL aliquots from each sample were transferred into a 96-well flat-bottom Microtest Plate (Sarstedt, Nümbrecht, Germany). For the sulfide assay (59), ferric ammonium sulfate (final concentration: 50 mM) and 4-amino-N,N-dimethylaniline sulfate (final concentration: 14.6 mM) were added, along with 100 µL of ultrapure water (0.05 µS cm^−1^, TOC < 5 ppb, Merck Millipore, Darmstadt, Germany) to reach a final volume of 250 µL. The plate was then incubated in the dark for 40 min, and absorbance was measured three times at 664 nm using an Infinite 200 Pro spectrophotometer (Tecan Life Sciences, Nänikon, Switzerland).

### Microbial methane production

Analysis of methane was conducted by gas chromatography (GC) with a barrier ion discharge detector (BID) using a helium plasma (Nexis GC-2030, Shimadzu Deutschland GmbH, Duisburg, Germany). The GC was equipped with a CP-Molsieve 5A capillary column (50 m × 0.32 mm × 30 µm, Agilent, Amstelveen, the Netherlands) using helium (5.0, AirLiquide, Oberhausen, Germany) as carrier gas at a column flow rate of 1.5 mL min^−1^. The GC oven temperature started at 180°C for 2 min, ramped with a rate of 10°C min^−1^ to 250°C, and held for 1 min. The injector temperature was set to 200°C with a split of 50% at a purge flow of 3.0 mL/min. The BID had a helium discharge gas flow of 70 mL/min, a sampling rate of 40 ms, and was tempered to 280°C. Total analysis time was 10 min with a retention time of hydrogen, oxygen, nitrogen, and methane of 3.72, 4.52, 4.86, and 5.71 min, respectively. Data were analyzed with the Lab Solutions Software (Version 5.92, Shimadzu Deutschland GmbH, Duisburg, Germany). The methane concentration measured in the headspace corresponds to the theoretical concentration in the liquid phase.

External calibration of methane concentrations was performed with self-made gas standards generated by injecting different volumes of pure methane (3.5, Air Liquide, Oberhausen, Germany) with a 1,000 µL gastight syringe (Hamilton, USA) into nitrogen-flushed vials.

Anoxic sampling of the microcosm headspace was performed with a 250 µL Hamilton syringe (Hamilton, USA) through the stopper of the microcosms and injection of 100 µL into the GC. Each microcosm headspace was analyzed three times. To avoid cross-contamination, the syringe was flushed with nitrogen (5.0, Air Liquide, Oberhausen, Germany) between sampling steps.

## RESULTS

**Fig 1:**
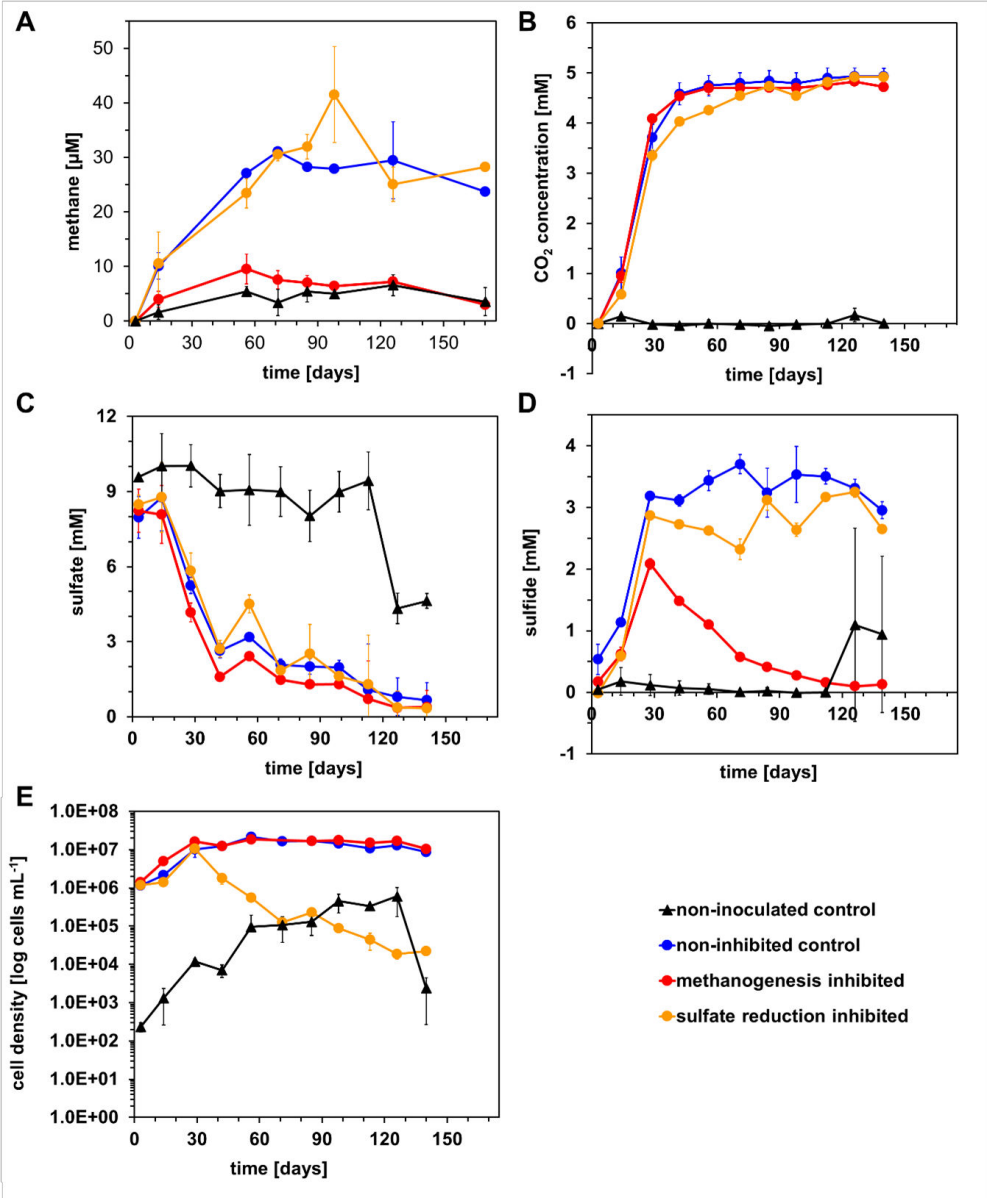
Crude oil degradation with sulfate reduction or methanogenesis as the terminal electron-accepting process. Non-inoculated control (black lines, contains only autochthonous microorganisms that might have survived autoclaving), non-inhibited control (blue lines), methanogenesis-inhibited with BES (red lines), and sulfate-reduction-inhibited with perchlorate and molybdate (yellow, lines). Perchlorate inhibition was from days 3 to 32, and molybdate inhibition from days 32 to 170. Data points depict mean values and standard deviations from triplicate incubations. (**A**) Development of methane production over time, (**B**) carbon dioxide evolution, (**C**) sulfate concentration, (**D**) sulfide concentration, and (**E**) cell density mL^−1^.

We investigated the coexistence of methanogenesis and sulfate reduction in a sulfate-adapted microbial community enriched from an oil reservoir, using a mixture of sodium molybdate and sodium perchlorate, or BES, as selective inhibitors. Molybdate and perchlorate inhibit sulfate reduction, while BES selectively inhibits methanogenesis due to its structural similarity to coenzyme M (CoM). Using crude oil as the sole carbon and electron source, we monitored the production of methane, carbon dioxide, and sulfide over time, along with sulfate depletion and cell growth (Fig. 1). In untreated controls and setups where sulfate reduction was inhibited, methane concentration steadily increased to 30 µM ± 1.2 over the first 85 days and then plateaued. This could be due to the presence of degradable fractions of crude oil that are not utilized by sulfate-reducing bacteria. The plateau after 85 days indicated that the carbon sources were probably depleted. The measured methane represented the theoretical concentration in the liquid phase, including all methane from the gas phase. This occurred alongside a sulfate reduction of 6 mM, indicating that active sulfate reduction did not suppress methanogenesis. In contrast, the BES-inhibited setup showed lower methane concentrations (9.5 µM ± 2.7), which were comparable to those in the uninoculated control (5.4 µM ± 0.9). Instead, it contained only the autochthonous microorganisms present in the added oil that survived the autoclaving process. Methane production rates were calculated based on the steepest concentration slopes between days 3 and 71, with the uninhibited control and sulfate-inhibited microcosms achieving the highest rates of 159 µM/a ± 3.3 and 157 µM/a ± 6, respectively, while methanogenesis-inhibited microcosms showed a four times lower rate of 39 µM/a ± 8.5. The produced carbon dioxide initially increased across all treatments, suggesting microbial metabolic activity, then plateaued at around 5 mM. The methanogenesis-inhibited setup showed similar carbon dioxide production as the control, resulting in the highest observed oil degradation rate with 76.4 mM ± 2.5 per annum [mM/a] CO_2_ development over the whole experiment. By comparison, the control without inhibitors and sulfate reduction-inhibited microcosms resulted in slightly lower carbon dioxide production rates of 66 mM/a ± 7.1 and 67.4 mM/a ± 2.1, respectively (Fig. 1B). Increased carbon dioxide production rates in the methanogenesis-inhibited setups suggested that sulfate reducers might have utilized BES as an additional carbon and electron source, but we did not support that with direct measurements of BES concentrations in the microcosms (60).

This pattern of carbon dioxide production was supported by sulfate depletion, showing an initial rapid decrease of 5.9 mM ± 0.5 sulfate in all microcosms within the first 40 days, which fitted well with the carbon dioxide evolution.

To estimate electron allocation, we assumed an average oxidation state of −1 for carbon in crude oil because the oil mostly consisted of alkanes (−2) and aromatics (0 to −1). For a carbon oxidation state of −1, a redox equation can be exemplarily calculated with benzene:


$$C_{6}H_{6}+12H_{2}O⟶6CO_{2}+30e^{-}+30H^{+}\;(oxidation\;half\;reaction)$$

$$SO_{4}^{2^{-}}+8e^{-}+10H^{+}⟶H_{2}S+4H_{2}O\;(reduction\;half\;reaction)$$

$$C_{6}H_{6}+3.75\;SO_{4}^{2^{-}}+7.5H^{+}⟶6CO_{2}+3.75\;H_{2}S+3H_{2}O\;(stoichiometric\;redox\;equation)$$

The total amount of electrons released from carbon oxidation is given by the produced CO₂, whereas the amount of electrons used for sulfate reduction is indicated by depleted sulfate. The difference is assumed to end up in biomass formation with a carbon oxidation state of 1.


$$30e^{-}\times c_{\left(CO_{2}produced\right)}=3.75\;\times 8e^{-}\times c_{\left(SO_{4}^{2^{-}}depleted\right)}+c_{\left(electrons\;in\;biomass\;built\;up\right)}$$

During the exponential phase within the first 40 days, measured carbon dioxide evolution (4.6 ± 0.2 mM, equivalent to 23.0 ± 1.0 mmol electrons/L) accounted for only ~54% of the electrons required for the observed sulfate reduction (5.3 ± 0.5 mM SO₄²⁻ = 42.4 ± 4.0 mmol electrons/L), resulting in an apparent electron deficit of 19.4 ± 4.1 mmol electrons/L. Electrons may have been routed through fermentative intermediates, such as H₂, acetate, or other volatile fatty acids, which can subsequently be oxidized by sulfate-reducing bacteria. Additionally, a portion of electrons was incorporated into microbial biomass.

In the methanogenesis-inhibited setups, sulfate reduction reached the highest rates with 84.5 mM/a ± 10.7, which might further indicate that BES served as a more easily available electron source for sulfate reducers (Fig. 1C). In total, based on the highest methane and carbon dioxide production observed in this experiment, 4 × 10^−2^ mol electrons/L (99.7%) were channeled to sulfate reduction, while 1.2 × 10^−4^ mol electrons/L (0.3%) were utilized for methanogenesis.

Sulfide production patterns followed those of sulfate reduction and carbon dioxide production, with lower total produced sulfide (2.8 mM ± 0.15) than expected from sulfate reduction (initial 9 mM), suggesting either losses during sampling and analysis or possible conversion to other sulfur compounds, such as polysulfide, that were missed in the sulfide analysis. This discrepancy suggests that not all reduced sulfate was recovered as free sulfide, which may be due to oxidation during sampling. Furthermore, sulfide may have precipitated as metal sulfides or been converted to other unmeasured sulfur species such as thiosulfate or polysulfides. While these explanations are plausible, we did not perform follow-up sulfur speciation analyses or mineral quantification to test these hypotheses, which limits our ability to conclusively determine the fate of the missing sulfur. The decline in sulfide concentrations after 30 days in BES-treated incubations remains unclear. While no trace metals were added, minor contamination from materials or the oil could have led to sulfide precipitation. Oxygen intrusion can be ruled out, as resazurin remained reduced. Continuous sulfate reduction (Fig. 1C) suggests ongoing sulfide production, but abiotic conversion of sulfide to other sulfur species, such as thiosulfate or polysulfides, might have occurred.

Cell growth, monitored by flow cytometry, was initially comparable across the non-inhibited controls and the sulfate reduction-inhibited setups (1.32 × 10^7^ cells/mL ± 3.08 × 10^6^). Then, molybdate addition to the sulfate reduction-inhibited microcosms at day 32 resulted in a continuous decrease in the cell counts, indicating inhibition by molybdate. Methanogenesis-inhibited setups initially showed higher cell density (1.63 × 10^7^ cells/mL ± 2.06 × 10^6^), aligning with the observations that this setup revealed the highest CO_2_-production and sulfate-reduction rates. In autoclaved controls, delayed microbial growth suggested the persistence of endospore-forming species like *Desulfotomaculum,* which is also visible in delayed sulfate reduction and sulfide production (Fig. 1E).

Overall, our findings show that methanogenesis and sulfate reduction can coexist in oil reservoir communities with crude oil as substrate and sulfate as electron acceptor.

### Microbial community composition

Variations in microbial communities across the different treatments were analyzed with amplicon sequencing of the V4 region of the 16S rRNA gene. The sequences were clustered into ASVs. The evaluation of the mock community revealed a nearly accurate community composition, highlighting the quantitative reliability of the amplicon sequencing method and confirming sequence cross-contamination of only 0.9%. The non-inoculated controls were sequenced, but after initial quality control and trimming, the remaining sequences were insufficient for analysis. This might be due to too low cell densities in the microcosms or potential DNA loss during the extraction and sequencing workflow.

**Fig 2:**
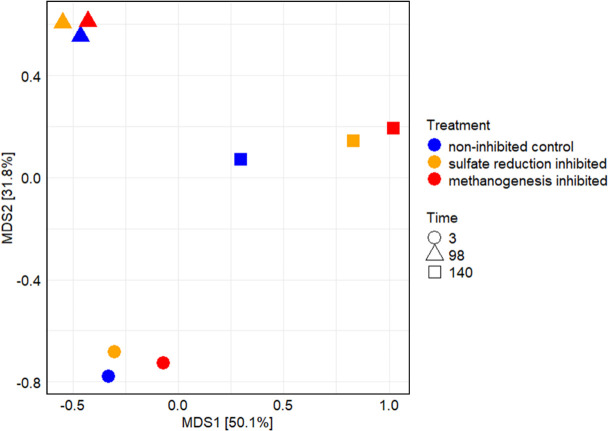
PCoA metric MDS plot of the Bray-Curtis dissimilarity of the microbial community compositions (average values of *n* = 3) of non-inhibited control (yellow), sulfate reduction-inhibited (green), and methanogenesis-inhibited (blue) microcosms at the beginning of the experiment 0 days (closed circles), after 98 days (closed triangles), and 140 days (closed squares) of incubation (stress = 0.089).

Bray-Curtis dissimilarities were calculated, which provided a quantitative measure of the differences in community composition between different treatments and time points. These dissimilarities were then visualized using a principal coordinates analysis (PCoA) metric multidimensional scaling (MDS) plot, illustrating the temporal shifts in microbial community structure over time (Fig. 2).

The Bray-Curtis dissimilarity analysis of the microbial community compositions showed clear clustering of the three treatments at each time point, indicating that the temporal development of the communities was more important than the differences between the treatments. With a longer incubation time, the communities differed more from each other, as displayed by the increased distances between the points of each cluster.

The Shannon index, a measure of community diversity that considers both species richness and evenness, was generally low but indicated slightly higher diversity in inhibitor-treated cultures. Specifically, diversity increased in sulfate reduction-inhibited (H = 2.68) and methanogenesis-inhibited (H = 2.90) cultures compared to the untreated culture (H = 2.28). This suggests that inhibitor treatment had a positive effect on biodiversity compared to the untreated control by either suppressing the dominant sulfate-reducers or providing another carbon source in the form of BES.

**Fig 3:**
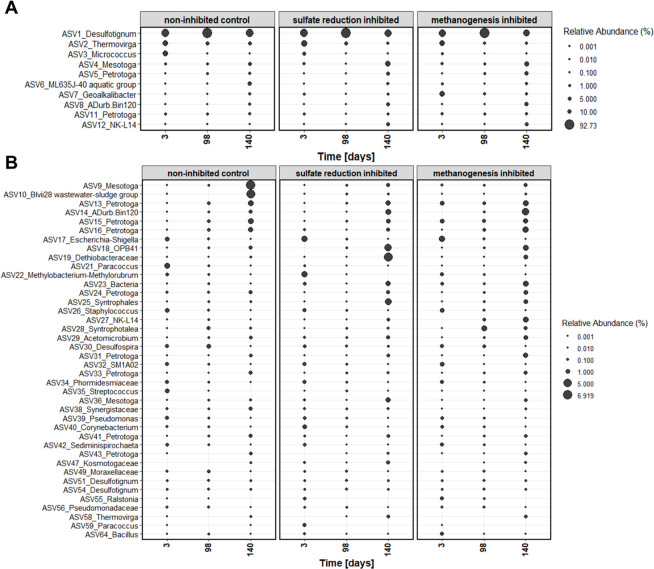
Relative abundance of the 50 most abundant ASVs in the non-inhibited control, sulfate reduction-inhibited, and methanogenesis-inhibited microcosms over 140 incubation days. Displayed taxa represent ASVs at the genus level; if classification to genus was not possible, the next higher taxonomic level is shown. (**A**) Ten most abundant ASVs and (**B**) most abundant ASVs 11–50. Consider different scales.

At the genus level, potential sulfate-reducing bacteria, such as *Desulfotignum* (ASV1), *Desulfospira* (ASV30), and *Geoalkalibacter* as part of the Desulfobacterota phylum (ASV7), dominated the bacterial communities, which was anticipated because we used a sulfate-adapted enrichment culture as inoculum. However, *Geoalkalibacter* species (e.g., *Geoalkalibacter ferrihydriticus*) are not primarily sulfate reducers but are iron-reducing or metal-respiring bacteria. *Geoalkalibacter subterraneus* strain *Red1* cannot actually reduce sulfate, sulfite, thiosulfate, glycine, fumarate, molybdate, chromium, or selenium (61). These organisms are not strictly oil-degrading microbes but are commonly detected in oil-associated environments and oil reservoirs (62, 63). Moreover, fermentative bacteria like *Thermovirga* (ASV2), *Mesotoga* (ASV4, 9, 36), and *Petrotoga* (ASV5, 11, 15, 16, 24, 31, 33, 41, 43) were the most prevalent ASVs, which are also important in oil reservoir communities (Fig. 3) (3, 64).

The first sample was taken 3 days after inoculation, which can explain the initial differences in the microbial communities, although one common inoculum was used. Both the control without inhibition and sulfate-reduction-inhibited treatments were dominated by *Desulfotignum* (ASV1), *Thermovirga* (ASV2), and *Micrococcus* (ASV3), which together accounted for over 80% of the microbial communities. In the methanogenesis-inhibited microcosms treated with BES, a distinct initial community emerged with *Geoalkalibacter* (ASV7) and *Mesotoga* (ASV4), as well as *Desulfotignum* (ASV1) and *Thermovirga* (ASV2). This indicated that the BES inhibition of methanogens immediately influenced the fermentative and sulfate-reducing community, while the perchlorate treatment appeared to affect only the *Micrococcus* (ASV3) population, promoting *Thermovirga* (ASV2). By day 98, *Desulfotignum* (ASV1) dominated all treatments, with around 90% relative abundance, even in the treatment where molybdate was added on day 32. Although cell counts dropped following molybdate addition, the sulfate reducer *Desulfotignum* continued to dominate.

After sulfate was depleted to 0.5 mM ± 0.1 on day 140, *Desulfotignum* declined, and the community shifted toward fermentative species, such as *Mesotoga* (ASV4), *Petrotoga* (ASV5), ML635J-40 aquatic group (ASV6), and *Thermovirga* (ASV2). When the most abundant ASV, *Desulfotignum* (ASV1), was excluded, more distinct patterns between the treatments emerged. In the non-inhibited microcosms, sulfate reducer populations decreased over time, while archaeal populations increased. This trend may be attributed to the low levels of sulfate available at the end of the experiment, leading to a shift in microbial activity. In the sulfate reduction-inhibited microcosms, sulfate reducer abundances were slightly lower than in the non-treated samples but still followed a similar trend. In the methanogenesis-inhibited microcosms, sulfate reducers appeared to decrease, but there was also a change in the relative abundance of the dominant ASVs, suggesting the presence of a different set of sulfate reducers compared to both the non-inhibited and sulfate-reduction-inhibited samples. This shift in microbial composition may indicate that BES addition supported a distinct community structure where sulfate reducers were affected differently than in the other treatments. Sulfate reducers may begin to die, producing necromass that serves as a substrate for other microorganisms, creating new ecological niches.

**Fig 4:**
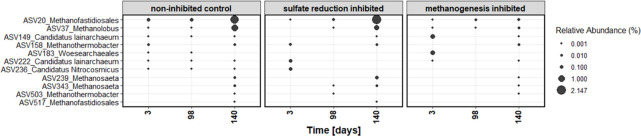
Relative abundances of archaeal ASVs over incubation time in the differently treated microcosms.

Among the archaea, seven ASVs were identified as potential methanogens, while five were most likely associated with other metabolic functions, such as syntrophic fermentation. Overall, the relative abundance of archaea within the total community remained low, with approximately 2.5% in both non-inhibited and sulfate reduction-inhibited microcosms after 140 days (Fig. 4).

The methanogenesis-inhibited microcosms exhibited lower relative abundances of archaea with less than 0.6%, showing that the inhibition of methanogens was effective, although not complete. Dominant organisms among the archaea were *Methanofastidiosales* (ASV20, 517) and *Methanolobus* (ASV37), both known methane producers. Although methane production was hindered by BES, both methanogens were still detected at lower abundances in the inhibited microcosms, indicating only partial inhibition of methanogenesis. By the end of the incubation, other methanogenic genera, such as *Methanosaeta* (ASV239 and 343) and *Methanothermobacter* (ASV503), emerged in all treatments, although with lower overall abundance. The observed methane production in the presence of sulfate-reducing bacteria might be mainly attributed to the most abundant *Methanofastidiosales* and *Methanolobus*, indicating that these methanogens were active despite the competitive presence of sulfate reducers.

## DISCUSSION

The terrestrial subsurface contains about 48% of Earth’s organic carbon, which can be converted to methane and carbon dioxide through fermentation and methanogenesis or to carbon dioxide via aerobic and anaerobic respiration with electron acceptors, such as NO₃⁻, SO_4_^2−^, Fe(III), Mn(IV), and others (65). However, electron acceptors for microbial respiration are commonly exhausted in the deep subsurface, leaving only fermentation and methanogenesis as processes for the degradation of organic matter. In oil production, sulfate-containing water is injected to maintain reservoir pressure and to enhance the oil recovery, which often introduces sulfate into the reservoir (3). Hence, sulfate-reducing bacteria can play a central role in hydrocarbon degradation after injection starts, with incomplete oxidizers converting organic compounds like lactate into acetate, and complete oxidizers fully mineralizing these compounds to carbon dioxide (6). These metabolic processes are influenced by oil composition, water availability, microbial activity, and thermodynamics, making oil reservoirs complex and heterogeneous ecosystems. While it’s generally accepted that sulfate-reducing bacteria outcompete methanogens, especially in sulfate-rich environments, evidence suggests that factors like limited or intermittent sulfate availability, spatial separation, or isolated water pockets might allow for their coexistence (3, 66). The coexistence of sulfate reduction and methanogenesis was shown in estuarine sediments, where sulfate concentrations up to 10 mM do not limit the onset or rates of methanogenesis (30). Methanogenesis is primarily regulated by substrate availability, allowing it to coexist with sulfate reduction across a broad spectrum of reduction rates. This might also be the case for oil reservoirs that are composed of highly organized and compartmentalized sediments.

### Coexistence of sulfate reduction and methanogenesis

This study demonstrates that the potential for metabolic coexistence persists even in a well-mixed system, although in minor amounts. Using a sulfate-amended culture from an offshore oil reservoir, we conducted incubation experiments with metabolic inhibitors. Our goal was to investigate the conditions under which methanogens and sulfate-reducing bacteria can coexist. These experiments provided insights into microbial competition and the cooperative degradation potential within oil reservoirs.

Our experiments showed that sulfate reduction did not inhibit methanogenesis, as both non-inhibited control and sulfate-reduction-inhibited setups produced similar levels of methane (30 µM). In contrast, estuarine sediments under sulfate-reducing conditions yielded higher methane (170–300 µM), most likely due to the more structured habitats and fresh *in situ* sampling (30). Hydrocarbon-contaminated soils produced up to 1 mM methane, which might again be due to the highly structured habitat compared to our well-mixed incubations (67).

### Coexistence mechanisms

Acetate, formate, and hydrogen are preferred substrates for both sulfate reduction and methanogenesis (9, 40, 68). Thermodynamically, sulfate reducers have a competitive advantage over methanogens by utilizing these substrates at lower concentrations, directing carbon flow toward carbon dioxide production rather than methane (15). This competition is often cited as the reason sulfate reduction restricts methanogenesis, especially in sulfate-rich environments (17, 18). The coexistence of sulfate reduction and methanogenesis under well-mixed incubation conditions in our study challenges the assumption of complete substrate exclusion by sulfate reducers. In contrast, substrate availability in sedimentary environments like oil reservoirs is often heterogeneous, creating localized niches where methanogens might persist. Spatial or temporal variations allow methanogens to access substrates not efficiently utilized by sulfate reducers. Partial substrate partitioning may also occur, with sulfate reducers dominating at low substrate concentrations, while methanogens thrive at higher concentrations or rely on alternative electron donors like methylamines or methanol. In environments with limited sulfate or reduced sulfate reduction rates, methanogens can further capitalize on decreased competition (30).

Similarly, methylotrophic methanogens found within the *Methanosarcinales*, *Methanobacteriales,* and *Methanomassiliicoccales* orders are capable of growth without a syntrophic partner. These organisms can be divided into two groups based on whether they possess cytochromes. Cytochrome-lacking methylotrophs depend entirely on hydrogen for methanogenesis, whereas those with cytochromes, such as certain *Methanosarcinales*, also utilize membrane-bound processes to oxidize methyl groups to carbon dioxide (69). Their metabolic process includes specialized methyltransferase systems, which channel methanol and methylated amines into the methanogenesis pathway at the stage of methyl-CoM (70). Here, the majority of methyl groups are reduced to methane, while a smaller portion is oxidized to carbon dioxide to produce reducing equivalents. Energy conservation occurs exclusively during membrane-bound methyl transfer because the membrane-bound methyltransferase functions in reverse, resulting in the dissipation of the proton or sodium motive force (70). Recent studies highlight the metabolic diversity of methylotrophic methanogens, including *Candidatus* Methanofastidiosa, which relies exclusively on methylated thiol reduction (71). A member of the order *Methanofastidiosales* (ASV 20) emerged as the most abundant archaeon in our incubation experiment. Genomic analyses reveal essential genes for their metabolism, such as those encoding the methyl-coenzyme M reductase complex and enzymes involved in the utilization of methanol (mtaA), methanethiol (mtsA), and methylamines (e.g., mtbA, mtmBC) (69, 72). In our enrichment cultures, the plateau in methane production may be attributed to the depletion of methoxylated substrates, which are critical for the activity of methylotrophic methanogens like *Methanofastidiosales* (ASV 20). However, since the presence and concentrations of methylated or methoxylated compounds in the oil were not analyzed, this explanation only represents a possible, but so far unconfirmed, mechanism. Although the oil composition in these cultures remains unclear, this substrate limitation could have constrained further methane production. While the relative abundance and genomic potential of *Methanofastidiosales* suggest a role in methane production, we emphasize that this link remains hypothetical in the absence of direct activity measurements. Future studies employing functional assays or stable isotope probing would be needed to confirm their contribution.

Other methanogens that do not require a syntrophic partner are, for example, *Candidatus* Methanoliparum, which was found in marine sediments and can grow with alkanes as a sole electron and carbon source, as well as methanogenesis as an electron-accepting process. Besides a complete methanogenesis pathway, it encodes a divergent methyl-coenzyme M reductase in the genome, which is supposed to activate alkanes (33, 35). Methanogens that do not rely on a syntrophic partner could be a good explanation for methanogenesis in the presence of sulfate reduction because they do not compete with sulfate reducers for hydrogen, formate, or acetate. Hence, the thermodynamic advantage of sulfate reducers does not come into play as long as there is enough oil present as substrate.

The metabolic coexistence of methanogenesis and sulfate reduction observed in this study was tested under consistent conditions. However, incomplete inhibition of sulfate-reducing bacteria in our experiments limited the full decoupling of these processes, which complicates the interpretation of the observed metabolic rates. In the literature, the coexistence of methanogenesis and sulfate reduction varies with environmental conditions, such as temperature, pressure, and substrate availability. In high-temperature, sulfate-limited settings like hydrothermal vents, methanogens, such as *Methanocaldococcus jannaschii,* can thrive due to the thermal limitations of sulfate-reducing bacteria (73, 74). Pressure and nutrient gradients also shape their distribution; for instance, deep biosphere studies show piezo-tolerant strains from both groups adapting to local geochemical niches (75, 76). While not universal, this metabolic coexistence is documented for hydrothermal systems, marine sediments, and subsurface habitats (77, 78). The presence of sulfate plays a crucial role in determining coexistence because sulfate-reducers can compete with methanogens for the substrates hydrogen and acetate. However, under sulfate-limited conditions, sulfate reducers can switch to fermentation, producing the same metabolites, acetate and hydrogen, which methanogens can then utilize to produce methane. This metabolic flexibility enhances their ability to coexist across different environments. While this coexistence is evident in oil reservoirs, its universality depends on environmental factors. Similar interactions may occur in other anaerobic ecosystems, such as marine sediments, landfills, and hydrothermal vents, where microbial communities must adapt to varying pressures, temperatures, and nutrient fluxes.

A possible mechanism for methanogenesis in the presence of sulfate reduction could also be direct interspecies electron transfer between fermenters and methanogens. For example, *Methanosarcina barkeri* can engage in interspecies electron transfer using conductive metals, which allows it to circumvent the competition with sulfate-reducing bacteria because no electron shuttles, such as hydrogen, formate, or acetate, are needed that could be used by the sulfate reducers (14). Nevertheless, sulfate-reducing organisms can also undergo direct interspecies electron transfer and again enter into competition for electrons with methanogens.

Here, methane production in the sulfate-enriched incubations was most likely driven by *Methanofastidiosales* and *Methanolobus*. Their abundances were reduced by BES treatment, although complete inhibition was not achieved. Additionally, two other methanogenic archaea, *Methanothermobacter* and *Methanosaeta*, were detected in all treatments at very low abundance, both of which are known methane producers. One reason for the coexistence of *Methanofastidiosales* and *Methanolobus* within sulfate-reducing communities may be due to their tolerance to elevated sulfide concentrations, which reached up to 3 mM in our microcosms. However, methanogens like *Methanobacterium* strain MOH have shown tolerance to sulfide concentrations as high as 20 mM (79). Other methane-producing archaea, such as *Methanosarcina barkeri,* remain metabolically active at sulfide levels around 7.5 mM, although with slower growth rates (80). Hence, sulfide is not a generic inhibitor of methanogens but might affect only specific species.

### Community composition

All taxonomic assignments were based solely on forward 16S rRNA reads due to the low quality of reverse reads. This may have reduced taxonomic resolution, particularly for closely related lineages, and could affect the precision of community interpretation. The microbial communities in our incubation experiments were dominated by sulfate reducers of the genus *Desulfotignum* from the phylum Desulfobacterota. The genus *Desulfotignum* often dominates sulfate-reducing communities in oil reservoirs with high sulfate levels. It is abundant in oil fields worldwide due to its ability to metabolize aliphatic organic acids commonly found in oil reservoirs (38). As shown in this work, most species of the genus are neither inhibited by sodium perchlorate nor sodium molybdate in commonly applied concentration ranges. The second most abundant ASV across all treatments was *Thermovirga*, a moderately thermophilic organism with a fermentative metabolism that utilizes amino acids, protein-based substrates, and certain organic acids, but not sugars, fatty acids, or alcohols. *Thermovirga* strain Cas60314T can reduce cysteine and elemental sulfur to sulfide, although it does not reduce thiosulfate (81, 82). The presence of *Thermovirga* indicates that, next to sulfur metabolism, fermentation is a main trait of the microbial community, which is typical for oil reservoirs (3).

Other than sulfate reducers and methanogenic archaea, fermenting bacteria were the most abundant groups within the different treatments. The most prominent members belonged to the ML635J-40 aquatic group (family *Bacteroidales*), *Petrotoga, and Mesotoga*. The role of the fermenters “ML635J-40 aquatic group” in anaerobic digestion remains largely unclear due to the lack of pure cultures. However, metagenomic analysis indicated that members of this group belonging to the Bacteroidetes phylum might play a significant role in hydrolyzing substrates during anaerobic digestion of the microalga *Spirulina* (83). *Petrotoga* species have been exclusively found in oil reservoirs (84), while *Mesotoga* species thrive in mesothermic, anoxic environments rich in hydrocarbons. *Mesotoga* produces little to no hydrogen and reduces sulfur compounds, growing better in the presence of thiosulfate, sulfite, and elemental sulfur (64, 85). Sulfate-reducing bacteria may promote *Mesotoga* growth by producing sulfite, similar to what we observed here. *Mesotoga* has been isolated from polluted marine sediments, oil reservoirs, and wastewater treatment plants, where it may also contribute to syntrophic acetate degradation (85).

Overall, the final microbial communities in this study exhibited low biodiversity with an average of H = 2.6 ± 0.3, with a slight increase observed in the inhibitor-treated incubations. Specifically, diversity in sulfate reduction-inhibited cultures (H = 2.68) and methanogenesis-inhibited cultures (H = 2.90) was higher than in the control without inhibition (H = 2.28). Possible reasons could be the introduction of BES as an additional, easily available substrate for sulfate reducers or the killing/inactivation of sulfate reducers by molybdate, which would release a plethora of new metabolites as substrates. Nevertheless, the Shannon index values were very low compared to microbial communities in other subsurface ecosystems, such as groundwater (e.g., H = 4.2) or typical oil reservoirs (H > 6) (86, 87). However, the low diversity is not due to a limited sequencing depth since we had 57,747 ± 18,339 (after filtering) reads per microcosm, which is far enough to cover the common biodiversity of microbiomes. According to the literature, sequencing depths of 10,000 to 15,000 reads per sample can already result in statistically stable diversity measures (88). Incubation time was the main driver influencing microbial activity and community composition in our incubations. This influence of the cultivation conditions is often overlooked in microcosm experiments, but is obviously a main driver of community compositions as soon as an environmental sample is incubated in the laboratory or cultivation conditions are changed.

### Inhibition effect

Our microcosms treated with BES to inhibit methanogenesis exhibited partial suppression, with initial methane concentrations peaking at 9.5 µM before declining. This decline may be attributed to BES hydrolysis, which produces metabolizable isethionate.

Despite inhibitor treatments, residual sulfate-reducing activity persisted, as evidenced by the dominance of *Desulfotignum* and sustained sulfate reduction rates. This incomplete suppression limits the extent to which sulfate reduction and methanogenesis can be fully decoupled in these experiments. Consequently, the interpretation of methane and CO₂ production rates must consider the complexity introduced by partial inhibitor efficacy. Additionally, the potential use of BES as a carbon source remains speculative due to the lack of direct measurements of BES concentrations or degradation products. Suppressed methanogenesis correlated with increased sulfate reduction and carbon dioxide production, likely due to stimulation of sulfate reducers (89). An alternative explanation for the increased sulfate-reducing activity in the methanogenesis-inhibited treatments is that sulfate reducers may have utilized BES or isethionates as an additional electron donor (60, 90). Sulfate reduction was also not completely inhibited upon addition of perchlorate or molybdate since, for example, *Desulfotignum* showed resistance to inhibitors, similar to earlier studies where species, such as *Desulfobacter curvatus* and *Desulfococcus multivorans,* exhibited partial molybdate resistance (43). While laboratory pure cultures can be effectively inhibited using various compounds, achieving complete inhibition of microorganisms within a complex community derived from an oil reservoir proves to be challenging.

### Limitations of the study

In our study, we mainly focused on the electron flow from the oxidation of oil to the terminal electron acceptors, sulfate or methanogenesis, to assess competition between the metabolic traits. Inhibitors were used to evaluate the electron fluxes to sulfate reduction or methanogenesis. However, this strategy only worked out to a certain extent because the inhibitors only partially reduced the observed degradation rates of either methanogenesis or sulfate reduction, and some sulfate reducers or methanogens were not affected. Furthermore, the used inhibitor for methanogenesis BES can also be used as a carbon and electron source by sulfate reducers, which might have artificially increased sulfate reduction rates. Nevertheless, the experimental setup clearly showed that methanogenesis took place in the presence of active sulfate reduction even under fully mixed conditions where the two metabolic traits got into a direct competition. However, since we could not analyze which trait consumes which substrates from the vast mixture of thousands of oil constituents, we could also not analyze if the coexistence of methanogenesis and sulfate reduction occurred because the two traits were both able to use hydrogen and acetate simultaneously, or if they also used different organic substrates and thus circumvented competition. Nevertheless, the growth of methylotrophic methanogens suggested that the latter avoidance of competition was one possible option.

In conclusion, our findings reveal that methanogenesis can persist in the presence of active sulfate reduction even in well-mixed incubations, challenging the traditional assumption that methane production is excluded under sulfate-reducing conditions in oil reservoirs. This coexistence may be facilitated by mechanisms, such as methylotrophy, which bypasses direct competition for substrates like hydrogen, carbon dioxide, and acetate, or alkane degradation by methanogens like *candidatus* Methanoliparum and Methanofastidiosales, or direct interspecies electron transfer. Additionally, the tolerance of methanogens to elevated sulfide concentrations likely supports their coexistence with sulfate-reducing bacteria within oil reservoir microbial communities.

## Data Availability

Raw sequencing reads have been deposited in the NCBI database under BioProject PRJNA1193503.
